# Chemistry beyond the Hartree–Fock energy via quantum computed moments

**DOI:** 10.1038/s41598-022-12324-z

**Published:** 2022-05-28

**Authors:** Michael A. Jones, Harish J. Vallury, Charles D. Hill, Lloyd C. L. Hollenberg

**Affiliations:** 1 School of Physics, University of Melbourne, Parkville, 3010 Australia; 2 School of Mathematics and Statistics, University of Melbourne, Parkville, 3010 Australia

**Keywords:** Physics, Quantum physics, Quantum simulation

## Abstract

Quantum computers hold promise to circumvent the limitations of conventional computing for difficult molecular problems. However, the accumulation of quantum logic errors on real devices represents a major challenge, particularly in the pursuit of chemical accuracy requiring the inclusion of electronic correlation effects. In this work we implement the quantum computed moments (QCM) approach for hydrogen chain molecular systems up to H$_6$. On a superconducting quantum processor, Hamiltonian moments, $\langle H^p\rangle$ are computed with respect to the Hartree–Fock state, which are then employed in Lanczos expansion theory to determine an estimate for the ground-state energy which incorporates electronic correlations and manifestly improves on the direct energy measurement. Post-processing purification of the raw QCM data takes the estimate below the Hartree–Fock energy to within 99.9% of the exact electronic ground-state energy for the largest system studied, H$_6$. Calculated dissociation curves indicate precision at about 10mH for this system and as low as 0.1mH for molecular hydrogen, H$_2$, over a range of bond lengths. In the context of stringent precision requirements for chemical problems, these results provide strong evidence for the error suppression capability of the QCM method, particularly when coupled with post-processing error mitigation. While calculations based on the Hartree–Fock state are tractable to classical computation, these results represent a first step towards implementing the QCM method in a quantum chemical trial circuit. Greater emphasis on more efficient representations of the Hamiltonian and classical preprocessing steps may enable the solution of larger systems on near-term quantum processors.

## Introduction

The computing resources required for the ab-initio solution of molecular systems generally scale exponentially as the system size increases, however, Feynman recognised that quantum computers may be able to solve these problems efficiently^1^. There has been considerable progress in developing quantum algorithmic approaches to the problem, and in understanding the quantum resources required for useful real-world cases^2–7^. Generally, because the underlying quantum algorithms are inherently phase sensitive, these approaches require fault-tolerant quantum error correction over hundreds of thousands to several million physical qubits to simulate molecular or condensed matter systems of scientific interest. With fault-tolerant quantum computation inaccessible in the short-to-medium term, approaches have been suggested that aim to make use of the advantages of quantum computation while keeping circuit depth minimal to reduce the accumulation of errors. Notable among these methods are variational hybrid algorithms such as the Variational Quantum Eigensolver (VQE)^8,9^ which exploits the quantum processor’s ability to efficiently encode the state of a quantum system while leveraging classical computation to optimise the state with respect to some inbuilt parameter set, using the expectation value of a chosen observable, usually the Hamiltonian, ${\mathcal {H}}$, as the cost function. Such algorithms have been considered as candidates^4,10^ for demonstrating quantum advantage^11^ on a problem of real scientific interest. Since the initial proposal^8^ and implementations of VQE^8,9,12,13^, various modifications and improvements to the algorithm, such as adaptive ansätze^14,15^ and alternative objective functions^16^ have been suggested.

While the reduced circuit depth of variational quantum algorithms provides a way to reduce errors, there is no way to completely prevent them on the Noisy Intermediate Scale Quantum (NISQ) hardware currently available^17^. As such, methods have been proposed to mitigate the effects of noise such as Richardson extrapolation^18,19^ and McWeeny purification^20,21^ among others. For molecular problems, these methods have achieved some success in effectively recovering the noise-free limit of the variational trial-state—usually constructed in the Hartree–Fock approximation^22^ as a first step. However, the real challenge remains to incorporate electronic correlation effects in a sufficiently noise-robust manner to break through the limitations of the Hartree–Fock trial-state and into the regime of chemical precision at the 1 kcal/mol (1.59 mH) level. While a more complicated trial state such as the UCC ansätz^23^ or its variations^24^ can, in principle, incorporate the required correlations; as the problem size is increased beyond what can be simulated classically the circuits soon become prohibitively long and inaccessible to near-term quantum devices.

**Figure 1:**
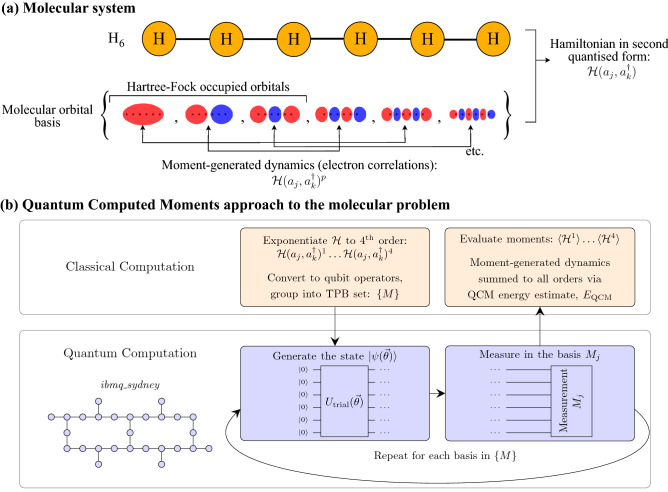
Overview of the quantum computed moments (QCM) approach applied to problems in chemistry. (**a**) The molecular system H$_6$ is represented by a second-quantised Hamiltonian over a set of molecular orbitals. The trial-state is the Hartree–Fock state, i.e. the occupation of the indicated orbitals. Application of the Hamiltonian moments allows for the generation of electronic correlation effects that the Hartree–Fock state cannot otherwise incorporate. (**b**) Overview of hybrid quantum/classical aspects of the QCM approach including a device map for the quantum processor *ibmq_sydney* used in this work.

Here we apply the recently introduced Quantum Computed Moments (QCM) approach^25^ to compute corrections to the conventional variational estimate, $\langle {\mathcal {H}}\rangle$, for molecular problems (defined by an electronic Hamiltonian, ${\mathcal {H}}$), on a superconducting quantum processor as outlined in Fig. 1 and discussed in further detail in the “Results” section. The QCM approach incorporates system dynamics through the computation of Hamiltonian moments, and uses results from Lanczos expansion theory^26^ to produce a dynamic correction, effectively summing these effects to all orders. The utility of the QCM method was previously demonstrated for quantum magnetism problems on a superconducting quantum processor of up to 25 qubits, providing stable estimates of the ground-state energy which improve on the corresponding direct energy measurements. Critically, the QCM results, even for this relatively large number of qubits, showed a high level of robustness to device errors and noise, suggesting utility for other quantum problems of interest on near-term devices. For our test molecular problem, we consider the ground state energy of hydrogen-atom chains up to H$_6$ computed with respect to a single-Slater determinant variational state.

Recently, other methods of using moments in the QC context have been proposed based on the power method^27^, extensions of the variational approach^28^, or the connected moments expansion (CMX)^29–32^. Unlike approaches such as the CMX, which applies Pade approximates to the *t*-expansion, the energy estimates obtained from Lanczos expansion theory are based on a rigorous diagonalisation of the Hamiltonian in Lanczos expanded form for a given finite moment order. In the past, direct comparisons show that the Lanczos expansion approach consistently provides a better energy estimate^33^.

These moment-based methods fit into a broader category of so-called Quantum Subspace Expansion (QSE) methods that classically diagonalise the Hamiltonian in an alternative basis where the diagonalisation can be carried out more efficiently/accurately. The generation of such a basis can be carried out by repeated application of the Hamiltonian operator to a suitably chosen trial-state (leading to moments-based methods) or by the application of other operators such as electronic excitations^34,35^, Pauli operators^36^ or matrix exponentials of the Hamiltonian^37,38^. Alternatively, a non-orthogonal basis can be defined as a more general set of states that can be prepared easily on the quantum processor^39–42^.

It is worth noting that Lanczos expansion theory is capable of more than calculating corrections to the ground state energy and that it should also be possible to calculate energy gaps^43^, thermodynamic properties and expectation values of physical quantities^44^ etc. Recently^45^ a simulated quantum computer was used to calculate Green’s functions based on the Hamiltonian moments and another method has been proposed^46^ for calculating exited states and time-evolution over a long time period from the Hamiltonian moments obtained from time-evolution over a short time period.

**Figure 2:**
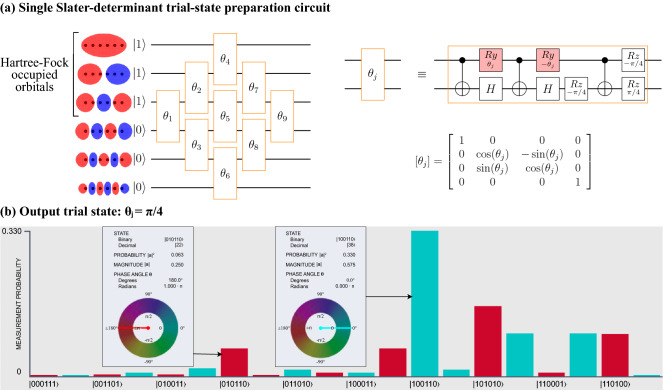
Trial state construction and implementation: illustrated case—the 6 atom hydrogen chain. (**a**) Qubit representation of the orbital basis, the trial circuit expressed in terms of Givens rotations^47–49^ and the Givens rotation expressed in terms of CNOT and single qubit gates and its matrix representation. Parameterised gates are shaded in red (**b**) The trial state produced when $\theta _j=\pi /4$, visualised using the Quantum User Interface (QUI) system^50^. Only the states representing the correct number of electrons are included on the horizontal axis since only these states can have non-zero amplitudes.

## Results

### Chemistry via Quantum Computed Moments

The QCM method is applicable to any molecular problem in general, however, for definiteness we consider linear chains of hydrogen atoms (Fig. 1a) governed by the usual second-quantised molecular Hamiltonian:

1 $$\begin{aligned} {\mathcal {H}}&=\sum _{jk}h_{jk}a^\dagger _ja_k+\sum _{jklm}g_{jklm}a^\dagger _ja^\dagger _ka_la_m, \end{aligned}$$

where $a^\dagger _j$ ($a_j$) are creation (annihilation) operators for an electron in molecular spin-orbital *j*. The one- and two-body molecular integrals ($h_{jk}$ and $g_{jklm}$ respectively) are computed efficiently on a classical computer and represent the kinetic energy of the electrons and the attractive electron-nuclear potential (*h*) and the repulsive electron-electron interactions (*g*). The Hamiltonian as written in Eq. (1) does not include the repulsion energy between the atomic nuclei—when calculating dissociation curves this must be taken into account as the nuclear repulsion varies as a function of atomic coordinates. This energy is referred to as the molecular energy. When performing optimisations for a single set of atomic positions the nuclear repulsion energy does not need to be taken into account as it is independent of the electronic configuration—this energy is referred to as the electronic energy. The problem is to use a quantum computer to find the ground-state energy to chemical precision (1 kcal/mol $\approx$ 1.59 mH). The conventional Variational Quantum Eigensolver (VQE) approach employs the quantum computer to compute the expectation value $\langle {\mathcal {H}}\rangle$, with respect to a well-chosen trial state, as a cost function in a classical minimisation loop. For chemical problems, the Hartree–Fock (HF) state is the chosen starting point for the minimisation of $\langle {\mathcal {H}}\rangle$. While gate-errors and device noise have a considerable effect on the values of $\langle {\mathcal {H}}\rangle$, error mitigation and purification techniques can essentially recover the HF energy^21^. In the quantum computation context, to go beyond the HF energy towards chemical precision one must include electronic correlations. While this can be achieved through the use of better trial state ansätz such as the Unitary Coupled Cluster ansätz (UCC)^23^ or alternative algorithms such as Quantum Phase Estimation (QPE)^51^, these approaches generally involve many qubits and/or deep circuits and/or quantum error corrected logical qubits and are therefore not suited to NISQ devices, even in the medium term. For NISQ devices to be of any use as a computational tool for chemistry, the algorithmic approaches must be adapted to be highly noise-robust in order to be capable of producing accurate results in the context of chemical precision.

The QCM method is based on an expansion of the Lanczos tridiagonal form in terms of moments $\langle {\mathcal {H}}^p\rangle$ (see section A in SI for details)^26^. The Hamiltonian moments encapsulate the system’s dynamics with respect to a given trial state—for the chemistry problems considered here, this equates to incorporating electronic correlations over the single Slater determinant trial state. In the quantum computing context, early explorations considered Hamiltonian moments in both adiabatic and gate-based circuit approaches^52^ and were later extended to the notion of direct computation of these quantities^53^. The resulting Quantum Computed Moments (QCM) method^25^ employs an approximation for the ground state energy in terms of connected moments (cumulants) $c_p$ of $\langle {\mathcal {H}}^p\rangle$, to fourth order, given by the expression^26,54,55^:

2 $$\begin{aligned} E_{\mathrm{QCM}}&\equiv c_1-\frac{c_2^2}{c_3^2-c_2c_4}\left( \sqrt{3c_3^2-2c_2c_4}-c_3\right) ,\nonumber \\ c_p&=\langle {\mathcal {H}}^p\rangle -\sum _{j=0}^{p-2}\left( \begin{matrix} p-1\\ j \end{matrix}\right) c_{j+1}\langle {\mathcal {H}}^{p-1-j}\rangle . \end{aligned}$$

The second term involving higher order connected moments not only provides a dynamical correction to the direct measurement result, $c_1\equiv \langle {\mathcal {H}}\rangle$, it also contributes a high degree of robustness to circuit errors.

For a given Hamiltonian, ${\mathcal {H}}$, the QCM method begins by exponentiating ${\mathcal {H}}$ to produce $\{{\mathcal {H}}^1, {\mathcal {H}}^2, {\mathcal {H}}^3, {\mathcal {H}}^4\}$. Here we keep ${\mathcal {H}}$ in second quantised form so the multiplications can be performed, for example, by using Wick’s theorem. After conversion to qubit operators, the growth of the number of terms in the exponentiated forms of ${\mathcal {H}}$ is controlled by forming tensor product basis (TPB) sets^9^ in a classical pre-processing step (as discussed in section D of the Supplementary Information). The trial state employed in this work is based on that used by Arute et. al.^21^, where each qubit represents the occupation state of a molecular orbital, classically pre-computed in the STO-3G minimal basis using the *Python* package *pyscf*^56^. (See section C in the Supplementary Information for additional details).

To reduce the computational burden on the quantum processor, an extension of a spin-symmetry reduction technique^21^ was employed to reduce the number of qubits by a factor of 2. While this qubit reduction (see section B in SI for details) requires several restrictions on the problem, most notably that the trial state must be a single Slater-determinant, the inclusion of electron dynamics introduced to the system by the QCM method allows the computation to achieve accuracy beyond what would normally be possible for such a trial state. This technique also allows for a reduction in the number of Tensor Product Basis (TPB) elements that need to be measured in the Hamiltonian averaging procedure by a factor of $N_s^{14}$ from the naive method of measuring the (worst case) ${\mathcal {O}}(N_s^{16})$ terms in ${\mathcal {H}}^4$ individually, where $N_s$ is the number of spin-orbitals. The results here were obtained using the conventional ${\mathcal {O}}(N_s^4)$ scaling of the Hamiltonian terms for which classical pre-processing on modest computing resources limited the molecular system size. However, our results together with the wide applicability of the QCM method to molecular systems in general, provides the tantalising possibility that with alternative Hamiltonian representations the QCM approach, coupled with error mitigation schemes, has the potential to provide accurate and strongly error-robust results for the ground state energy of larger chemical systems on near-term quantum computers.

**Figure 3:**
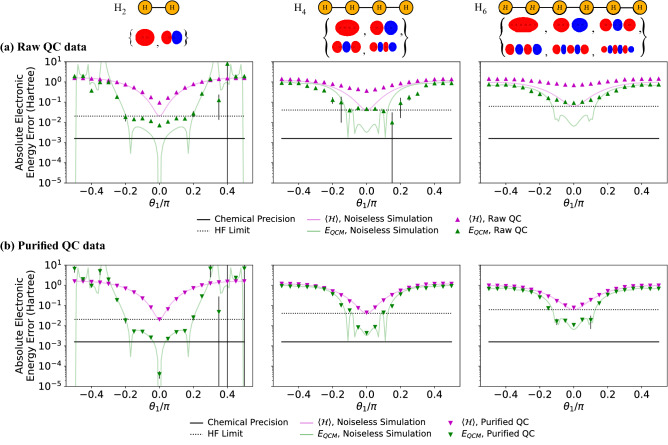
Results of varying the leading circuit parameter, $\theta _1$ for (from left to right) H$_2$, H$_4$, H$_6$. The vertical axis is the absolute error in electronic energy from the FCI result $|E-E_\mathrm{FCI}|$ on a logarithmic scale. Ideal simulations were performed without errors or shot noise while QC data was averaged over 4 runs (total 8192 shots). Statistical error bars on the QC data represent one standard deviation and are often smaller than the data points. (**a**) Comparison of raw data results to noiseless simulation. (**b**) Comparison of purified data to noiseless simulation.

**Figure 4:**
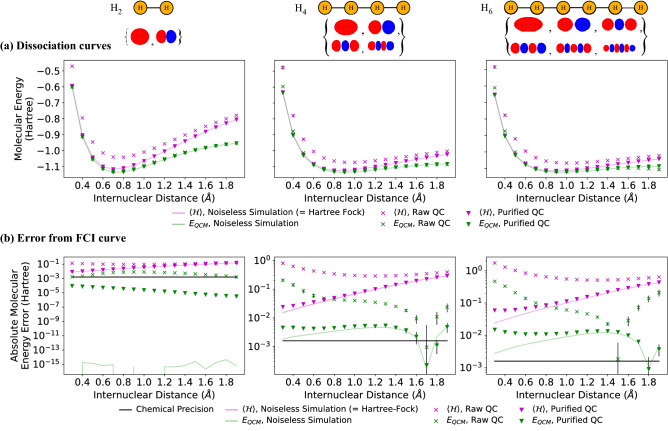
Dissociation curves for hydrogen chains. The energy values at each point correspond to a single energy evaluation (8192 shots) performed at parameter value $\theta =(0,0,\dots )$. (**a**) Molecular energy as a function of inter-nuclear distance. The purple line is the noiseless simulation result which reproduces exactly the Hartree–Fock energy. (**b**) The absolute value of the error in molecular energy relative to the FCI results.

### Circuit parameter sweep

With respect to the single Slater-determinant trial-state defined in Fig. 2 (parameter set $\mathbf {\theta }$), all observables contained in the TPB sets were measured directly on the *ibmq_sydney* device for the molecular systems H$_2$, H$_4$ and H$_6$ with all bond lengths set to 0.74 Å (the equilibrium bond distance of molecular hydrogen). Each TPB set was measured using 8192 shots. To illustrate variational behaviour we present the data for values of the opening trial-state parameter $\theta _1$ around the Hartree–Fock state compared to the Hartree–Fock energy and the Full Configuration Interaction (FCI) results in Fig. 3a (see section F in the Supplementary Information for ensemble data over the variational parameter sets). The error robustness of the QCM results is quite evident for all three molecular systems—while the pink triangles representing the direct measurement result (with respect to $\theta _1$) move significantly away from the solid pink line indicating the trial-state limit (HF energy), the upward shift of the QCM due to device errors (green triangles shifting away from the green line) is suppressed by an order of magnitude. Despite the large number of observables required to be measured on the QC device in the determination of the moments, the QCM correction term consistently suppresses the device errors contained in the uncorrected computation $c_1 = \langle {\mathcal {H}}\rangle$. In fact, for the largest system, H$_6$, the raw QCM data already recovers 99.7% of the minimised trial-state energy at the HF point.

### Density matrix purification

To investigate the potential of the method to reach beyond the Hartree–Fock energy we perform McWeeny purification on the 1-body reduced density matrix (1-RDM)^20^—this procedure has been shown to improve the direct energy measurement result at the Hartree–Fock point^21^. Details on the purification procedure can be found in section E in the Supplementary Information. The results of the RDM purification procedure are shown in Fig. 3b. As observed in previous work^21^, the purification procedure applied to the direct energy measurement (pink inverted triangles) goes a long way to recovering the trial-state energy (pink line). However, the key observation is that the RDM purification is also highly effective in correcting the remaining device errors in the QCM calculation (green inverted triangles), explicitly recovering a portion of the electron correlation and pushing the result beyond the HF energy (black dashed line).

The high level of accuracy (relative to the FCI result) in the H$_2$ case is in part due to the fact that the Hilbert space spanned by the minimal basis is reasonably small, so the Hartree–Fock state is a better approximation to the true ground-state. For the H$_4$ and H$_6$ cases the accuracy of the Hartree–Fock method is decreased and so the QCM results based on the HF state are also reduced in accuracy. Systematic improvement of the trial state (of the form usually used in VQE calculations) is expected to allow the QCM method to more accurately estimate the ground-state energy.

### Dissociation curves

Finally, we repeat the calculations at a range of atomic separations to compute the dissociation curves shown in Fig. 4. As expected, in the absence of noise, the QCM correction (green line) incorporates the electronic correlations at a sufficient level to recover energies below the Hartree–Fock energy (pink line) for all bond distances examined for all three molecules. For most cases, especially at longer bond lengths where the Hartree–Fock energy diverges more significantly from the FCI results, the moments-based correction without any additional error mitigation (green crosses) is sufficient to calculate energies below the Hartree–Fock energy, even when performed on a noisy quantum device.

With the application of 1-RDM purification, the moments based method on the quantum device *ibmq_sydney* (green inverted triangles) was able to outperform the noiseless Hartree–Fock results for all molecular geometries considered, reaching error thresholds relative to the FCI results of order 10 mH for the longest chain, H$_6$, and 0.1 mH for molecular hydrogen. The latter is below the chemical precision threshold of 1.59 mH (black line in Fig. 4b). We note that this result is within chemical precision of the FCI energy calculated in the minimal STO-3G basis and that a significantly larger basis is required to claim true chemical accuracy^4^. We also note that the results presented by Arute et. al.^21^ are likewise restricted by the choice of basis and are in fact within chemical precision only of the Hartree–Fock energy and not the FCI ground state. Additionally, the results presented here do not rely on the symmetry of the system and could, in principle, be calculated for any spatial arrangement of the atoms. This is in contrast to the results of Kawashima et. al.^57^ where chemical precision relative to FCI calculations was achieved for the H$_{10}$ ring by exploiting the high level of rotational symmetry in the system. Chemical precision has also been reported for NaH^58^ on 4 superconducting qubits using a frozen core approximation and a UCC inspired circuit, a problem of similar size to the H$_2$ molecule simulated, reaching chemical precision relative to the FCI energy. Another work^59^ simulates H$_2$O on 11 trapped-ion qubits by carefully selecting the excitation operators to be included in a UCC-style ansätz circuit and achieves errors relative to the ideal result and standard deviations at the same order as chemical precision for the first few determinants. Given the precision of the QCM method when performed using the less accurate single Slater-determinant trial state it is expected that application of the method to UCC-style ansätz circuits would provide a further improvement on the precision of the ground-state energy estimates.

## Discussion

We have applied the Quantum Computed Moments method to the quantum-chemical problem of computing the ground state energy of linear chains of hydrogen atoms and found that, even for restrictive single Slater-determinant trial states, use of the QCM method allows for recovery of electron correlation and therefore of energies below the Hartree–Fock threshold. Though computation of the Hamiltonian moments may seem expensive, the scaling of the number of terms in ${\mathcal {H}}^4$ is significantly better than the worst-case as seen in section D of the SI and could be further reduced by transformation into an alternate basis. Additionally the number of measurements can be controlled by grouping mutually commuting operators for simultaneous measurement.

With the addition of McWeeny purification, we demonstrate that the method is capable of outperforming (noise-free) Hartree–Fock calculations, even when the moments are computed on noisy present-day quantum hardware, for chains of up to 6 atoms, the largest system studied here requiring up to 27 CNOT gates. For molecular hydrogen, H$_2$, we achieve results that are within chemical precision of the FCI result calculated with respect to the minimal basis set for a range of inter-nuclear distances around the equilibrium bond length. For the H$_6$ chain at 0.74 Å, with no error mitigation the QCM method is able to recover 97.1% of the molecular energy while the usual direct measurement of $\langle {\mathcal {H}}\rangle$ is able to return only 78% of the energy due to noise in the trial circuit preparation.

Although the McWeeny purification technique is not easily generalised to states that cannot be represented as a single Slater-determinant, the QCM method performs well even without the purification and would likely benefit from any improvement in the trial state. Such improvement could come in the form of a UCCD circuit ansatz, for example, and should allow the QCM result to approach the FCI energy more closely while maintaining the QCM method’s resilience to noise. Experimentation with improved circuit ansatze is left to future work as is a detailed time-cost analysis. Furthermore it is possible to adapt the QCM method for the computation of excited state energies^43^ and to properties other than the energy^44^. With improvement of the trial state, combined with error mitigation techniques and alternative Hamiltonian representations to control the scaling of the problem the QCM method is a promising technique for the pursuit of chemical accuracy on present-day quantum hardware.

## Methods

### Spin-degeneracy qubit-reduction

Due to the spin-symmetry of the system the number of qubits required for quantum simulation can be reduced by a factor of 2 if the trial state is restricted to a single Slater-determinant, $|\Psi _P\rangle$ (see section B in SI). Given the restricted trial state it is possible to extract expectation values for any excitation operator from the 1-body reduced density matrix (1-RDM) according to the equation

3 $$\begin{aligned} \langle \Psi _P|a^\dagger _ja^\dagger _k\dots a_{k'}a_{j'}|\Psi _P\rangle&=\left| \begin{matrix} \langle a^\dagger _ja_{j'}\rangle &{}\langle a^\dagger _ja_{k'}\rangle &{}\cdots \\ \langle a^\dagger _ka_{j'}\rangle &{}\langle a^\dagger _ka_{k'}\rangle &{}\cdots \\ \vdots &{}\vdots &{}\ddots \end{matrix}\right| . \end{aligned}$$

The qubit-reduction method is described in more detail in section B in the Supplementary Information.

### The chemical Hamiltonian

The chemical Hamiltonians used in this work were computed using the *python* package *pyscf*^56^ to optimise the molecular orbitals and were converted to qubit Hamiltonians using the Jordan-Wigner transform^60^.

### Measurement details

To reduce the required number of state preparations, the Pauli strings required for measurement of the 1-RDM are grouped into ${\mathcal {O}}(N_s^2)$ mutually commuting tensor product basis sets^9^ as described in section D in the Supplementary Information. For each TPB, the trial circuit was executed and the output state was measured 8192 times to obtain the average results. To estimate uncertainties, the 8192 measurement results were randomly assigned to one of 4 bins. Each bin (of roughly 2000 results each) was processed individually and the standard deviation of these 4 results was calculated. Quantum computed data for the graphs in Figs. 3 and 4 were taken from the device *ibmq_sydney* and simulated results were calculated using a statevector simulator without shot noise. Dissociation curves were also calculated on the *ibmq_toronto* and *ibmq_guadalupe* devices and found to be consistent with the *ibmq_sydney* results.

### Density matrix purification

Purification of the 1-body reduced density matrix was performed following previous works^20,21^ by iteratively applying the equation:

4 $$\begin{aligned} R_{j+1}=3R_j^2-2R_j^3, \end{aligned}$$

where $R_0$ is the unpurified matrix and $R_j$ is the matrix after *j* iterations of the purification procedure. The purification method is described in more detail in section E of the Supplementary Information.

## Supplementary Information

Supplementary Information.

## Data Availability

The data that support the findings of this study are available from the corresponding author upon reasonable request.
