# Supplementary material for: Chemistry beyond the Hartree–Fock energy via quantum computed moments
Source: Sci Rep. 2022 May 28;12:8985. doi: 10.1038/s41598-022-12324-z (PMC9148318; doi:10.1038/s41598-022-12324-z)
Supplement: Supplementary file 1 — Supplementary Information. [file 41598_2022_12324_MOESM1_ESM.pdf]

Chemistry beyond the Hartree-Fock energy via quantum computed moments

Michael A. Jones[1], Harish J. Vallury[1], Charles D. Hill[1,2], Lloyd C. L. Hollenberg[1,*]

[1] *School of Physics, University of Melbourne, Parkville 3010, AUSTRALIA*

[2] *School of Mathematics and Statistics, University of Melbourne, Parkville 3010, AUSTRALIA*

[*] *Corresponding author. email: lloydch@unimelb.edu.au*

Supplementary Information

A. The Quantum Computed Moments approach

We outline the background to the QCM method as follows. For a given Hamiltonian, the Lanczos recursion tri-diagonalises the system starting with an appropriate initial trial-state, $|v_1\rangle$,

$$|v_{i+1}\rangle = \frac{1}{\beta_i}\left[(\mathcal{H}-\alpha_i)|v_i\rangle - \beta_{i-1}|v_{i-1}\rangle\right], \qquad \text{(SI.1)}$$

where $\alpha_i = \langle v_i|\mathcal{H}|v_i\rangle$ and $\beta_i = \langle v_{i+1}|\mathcal{H}|v_i\rangle$ are the diagonal and off-diagonal elements of the tri-diagonal matrix respectively [62]. By introducing the cumulants, c_p [26, 54], of the Hamiltonian with respect to the trial state $|v_1\rangle$,

$$c_p = \langle \mathcal{H}^p\rangle - \sum_{j=0}^{p-2} \binom{p-1}{j} c_{j+1}\langle \mathcal{H}^{p-1-j}\rangle, \qquad \text{(SI.2)}$$

a general expansion for the matrix elements was uncovered [26],

$$\begin{aligned}\alpha(z) &= c_1 + z\frac{c_3}{c_2} + z^2\frac{3c_3^2 - 4c_2c_3c_4 + c_2^2c_5}{4c_2^4} + \ldots,\\ \beta^2(z) &= zc_2 + z^2\frac{c_2c_4 - c_3^2}{2c_2^2} + \ldots. \end{aligned} \qquad \text{(SI.3)}$$

Here the continuous, positive parameter z is related to the recursion index [26, 33, 63]. These general expressions for the matrix elements allow for the expression of the ground state energy in terms of an infimum theorem [33];

$$E_0 = \inf_{z>0}\left[\alpha(z) - 2\beta(z)\right]. \qquad \text{(SI.4)}$$

Truncating the z-expansion in terms of the moments to $p_{\max} = 4$ produces the expression for the ground state energy approximate used in the QCM method [55],

$$E_{\mathrm{QCM}} \equiv E_0^{(4)} = c_1 - \frac{c_2^2}{c_3^2 - c_2c_4}\left(\sqrt{3c_3^2 - 2c_2c_4} - c_3\right), \qquad \text{(SI.5)}$$

where the first term is simply $c_1 = \langle\mathcal{H}\rangle$ and the second term, depending on the higher order moments, provides a correction to this energy. In practice the moments-corrected energy estimates are found to be non-variational, likely due to the truncation to moments of order 4. In the limit of infinite moments, the tri-diagonal Hamiltonian would be expected to approach the full Hamiltonian and variational behaviour would be recovered.

B. Spin-degeneracy qubit reduction

Although trial state depth is potentially the most precious quantum resource in a VQE calculation, any reduction in the number of qubits required for the simulation, such as through qubit tapering [64], will allow reduction of the circuit depth since fewer gates will be needed to generate a sufficiently entangled quantum state over the reduced number of qubits. In addition, any reduction in the number of qubits will likely decrease the number of measurements and therefore, the number of trial state preparations required per energy estimation.

There are several properties of the systems studied here, the linear hydrogen atom chains, that allow the reduction technique used in [21] to be applied. It should be noted that these restrictions apply only to the qubit-reduction technique not to the QCM method in general. The restrictions are;

- The Hamiltonian of the system is not explicitly dependant on spin, while this is true for all molecules in a vacuum, it is not true in the presence of an external magnetic field, which could potentially limit the applicability of this technique.
- The trial state used to simulate the system consists of a single Slater determinant. The Slater determinant is often used in quantum chemistry as a starting point from which more accurate states can be constructed. However, as the objective of the QCM method is to reduce the complexity of the trial state, the use of a single determinant trial state is a reasonable choice. Additionally, it is

seen that the use of the QCM method allows for the recovery of energies below the Hartree-Fock energy.

- The system is simulated in a singlet state, while this can be done for any molecular system, the ground state of paramagnetic materials, such as oxygen gas (O_2), is not a singlet. In these cases, application of the qubit reduction would lead to reduced accuracy.

The Slater determinant trial state can be written in second-quantised form as;

$$|\Psi_{\text{trial}}\rangle = |\psi_P\rangle = \prod_{p\in P} b_p^\dagger|0\rangle, \tag{SI.6}$$

where P is a list of the basis states from which the determinant is constructed, $b_p^\dagger$ is the fermionic creation operator for state p and $|0\rangle$ is the vacuum state. In this form, the antisymmetrisation of the state is encoded in the anticommutation of the fermionic operators. The single excitation operator $b_q^\dagger b_r$ will have vanishing expectation value with respect to the trial state unless $qr \in P$, in which case

$$\begin{aligned}\langle\Psi_P|b_q^\dagger b_r|\Psi_P\rangle &= \delta_{qr}\langle\Psi_P|\Psi_P\rangle - \langle\Psi_P|b_r b_q^\dagger|\Psi_P\rangle \\ &= \delta_{qr},\end{aligned} \tag{SI.7}$$

where the last step uses $q \in P$ and $(b_q^\dagger)^2 = 0$ to show that the second term vanishes. From this point on, it will be assumed that expectation values are taken with respect to the trial state $|\Psi_P\rangle$. Using the anticommutation relations it can similarly be shown that the expectation value of a double-excitation operator is;

$$\langle b_q^\dagger b_r^\dagger b_{r'} b_{q'}\rangle = \delta_{qq'}\delta_{rr'} - \delta_{rq'}\delta_{qr'} = \begin{vmatrix} \delta_{qq'} & \delta_{qr'} \\ \delta_{rq'} & \delta_{rr'} \end{vmatrix}. \tag{SI.8}$$

and an arbitrary level excitation operator has the expectation value;

$$\langle b_q^\dagger b_r^\dagger b_s^\dagger \dots b_{s'} b_{r'} b_{q'}\rangle = \begin{vmatrix} \delta_{qq'} & \delta_{qr'} & \delta_{qs'} & \cdots \\ \delta_{rq'} & \delta_{rr'} & \delta_{rs'} & \cdots \\ \delta_{sq'} & \delta_{sr'} & \delta_{ss'} & \cdots \\ \vdots & \vdots & \vdots & \ddots \end{vmatrix}. \tag{SI.9}$$

It is worth noting that;

- Swapping two creation (annihilation) operators is equivalent to swapping two rows (columns) in the determinant. Both actions introduce a factor of -1 as expected.

- If two creation (annihilation) operators act on the same state, then two rows (columns) in the determinant are equal and both sides of the equation vanish.

- If a creation (annihilation) operator does not act on a state in the Slater determinant, the equation does *not* hold. In this case, a modified form of the equation is required;

$$\langle b_q^\dagger b_r^\dagger \dots b_{r'} b_{q'}\rangle = \begin{vmatrix} \langle b_q^\dagger b_{q'}\rangle & \langle b_q^\dagger b_{r'}\rangle & \cdots \\ \langle b_r^\dagger b_{q'}\rangle & \langle b_r^\dagger b_{r'}\rangle & \cdots \\ \vdots & \vdots & \ddots \end{vmatrix}. \tag{SI.10}$$

When all indices are $\in P$, equation SI.7 reduces this to equation SI.9, but if any creation (annihilation) index is $\notin P$, then every entry in that row (column) will be 0 and the determinant will vanish, as required.

To allow for a more general state preparation, the basis states used in the construction of the Slater determinant are allowed to vary from the basis in which the second-quantised Hamiltonian is written. The trial state basis will be represented by the creation (annihilation) operators $b^\dagger$ (b) while the Hamiltonian basis will be represented by the operators $a^\dagger$ (a). The relation between these bases can be written as;

$$a_j^\dagger = \sum_q A_{jq} b_q^\dagger, \tag{SI.11}$$

for appropriately normalised complex amplitudes A_{jq}. The expectation value for an excitation operator in the Hamiltonian basis is then

$$
\begin{aligned}
\langle\Psi_P|a_j^\dagger a_k^\dagger\dots a_{k'}a_{j'}|\Psi_P\rangle &= \sum_{qr\dots r'q'} A_{jq}A_{kr}\dots A^*_{k'r'}A^*_{j'q'}\langle\Psi_P|b_q^\dagger b_r^\dagger\dots b_{r'}b_{q'}|\Psi_P\rangle, \\
&= \sum_{qr\dots r'q'} A_{jq}A_{kr}\dots A^*_{k'r'}A^*_{j'q'}\begin{vmatrix} \langle b_q^\dagger b_{q'}\rangle & \langle b_q^\dagger b_{r'}\rangle & \cdots \\ \langle b_r^\dagger b_{q'}\rangle & \langle b_r^\dagger b_{r'}\rangle & \cdots \\ \vdots & \vdots & \ddots \end{vmatrix}, \\
&= \begin{vmatrix} \sum_{qq'} A_{jq}A^*_{j'q'}\langle b_q^\dagger b_{q'}\rangle & \sum_{qr'} A_{jq}A^*_{k'r'}\langle b_q^\dagger b_{r'}\rangle & \cdots \\ \sum_{rq'} A_{kr}A^*_{j'q'}\langle b_r^\dagger b_{q'}\rangle & \sum_{rr'} A_{kr}A^*_{k'r'}\langle b_r^\dagger b_{r'}\rangle & \cdots \\ \vdots & \vdots & \ddots \end{vmatrix}, \\
&= \begin{vmatrix} \langle a_j^\dagger a_{j'}\rangle & \langle a_j^\dagger a_{k'}\rangle & \cdots \\ \langle a_k^\dagger a_{j'}\rangle & \langle a_k^\dagger a_{k'}\rangle & \cdots \\ \vdots & \vdots & \ddots \end{vmatrix}.
\end{aligned} \tag{SI.12}
$$

Since the Hamiltonian contains only single- and double-excitations, the qubit reduction as applied in [21] required only the expression for double-excitation operators, which had previously been determined in [20]. The more general form here is required for use with the Hamiltonian moments which can include up to 8th level excitation operators.

Equation SI.12 allows the measurement of an ith level excitation operator to be reduced to the measurement of i^2 single-excitation operators and the calculation of the determinant of an $i \times i$ matrix (as previously derived in [65]). Since the system is being simulated in the singlet state and the Hamiltonian does not depend explicitly on the spin, any spin crossing 2-mode terms must have vanishing expectation values and all down-spin terms will have expectation values identical to the corresponding spin-up terms:

$$
\begin{aligned}
\langle a^\dagger_{(j,\uparrow)}a_{(k,\downarrow)}\rangle &= 0, \\
\langle a^\dagger_{(j,\uparrow)}a_{(k,\uparrow)}\rangle &= \langle a^\dagger_{(j,\downarrow)}a_{(k,\downarrow)}\rangle.
\end{aligned} \tag{SI.13}
$$

Using equations SI.12 and SI.13 any expectation value required for estimating the Hamiltonian can be calculated by simulating and measuring only the spin-up states (or equivalently only the spin-down states), allowing the number of qubits required for simulation to be reduced by a factor of 2.

While this procedure may seem expensive at first glance, it is important to note that the level of the expectation values and therefore the size of the determinants to be computed is dependant only on the level of the moments correction used and not on the system size. Additionally, there are only $N_s^2/4$ single-excitation expectation values from which the determinants can be constructed, for a simulation involving N_s spin-orbitals. Each of these expectation values only needs to be measured once and section D discusses ways in which the number of trial state preparations can be reduced further. From these $\mathcal{O}(N_s^2)$ expectation values, the 1-body reduced density matrix can be constructed and all non-vanishing fermionic expectation values can be written as minors (determinants of a submatrix) of the 1-RDM.

C. The trial-state

When applying the VQE algorithm to examples from quantum chemistry it makes sense to map the molecular orbitals, constructed as linear combinations of atomic orbitals (LCAOs), to each qubit. The molecular integrals of Equation 1 can then be calculated with respect to these orbitals. In this work, the STO-3G basis is used to represent each atomic orbital by a linear combination of 3 Gaussian orbitals, the molecular orbitals and their corresponding integrals are then calculated using the python package *pyscf* [56] through the *qiskit* package [66]. Because the molecular orbitals are pre-optimised by *pyscf*, the solution to the Hartree-Fock minimisation should trivially be the occupation of the η lowest energy spin-orbitals, where $\eta = N_s/2$ (for the hydrogen atom chains) is the number of electrons. However if the molecular orbitals were not pre-optimised (for example, the use of alternate bases can reduce the number of terms in the Hamiltonian, see Appendix D) then the optimal trial circuit parameters will need to be found. The trial circuit used here is based on that used in [21].

From the initial state, a series of parameterised Givens rotations [47] is applied between neighbouring qubits following the optimal layout determined in [48]. The resulting circuit (see Figure 2) has depth $\mathcal{O}(N_s)$ and gate count $\mathcal{O}(N_s^2)$. The rotations used here are de-

fined in such a way that when the gate parameter, θ, goes to 0, the Givens rotation reduces to the identity operation and therefore a circuit with the parameter set $(0,0,0,\dots)$ will return the classically pre-optimised Hartree-Fock state.

D. Problem scaling

While the QCM method provides a technique for the suppression of noise for VQE, which in turn was developed to take advantage of near-term quantum hardware while reducing the impact of errors, a possible limitation of VQE and, by extension, the QCM method is the potentially rapid growth of the number of terms present in the Hamiltonian which determines the number of measurements and hence the number of state preparations required of the quantum processor. In the usual molecular orbital basis the number of terms in the quantum chemical Hamiltonian scales as $\mathcal{O}(N_s^4)$ (where N_s is the number of spin-orbitals) which is then amplified by the exponentiation required for the moments correction. The scaling problem in VQE has led to various methods designed to reduce the number of measurements required. These can be split into two categories. The first class of methods are those that aim to reduce the number of terms in the Hamiltonian, for example by transforming the operator into a different basis in which the number of terms scales better than the usual $\mathcal{O}(N_s^4)$ [67–69] or by neglecting Hamiltonian terms with a weight below a certain threshold, such as interaction terms between spatially separated wavefunctions (orbitals). The second class of methods are those that aim to reduce the number of measurements (but not the number of terms) by partitioning the Hamiltonian terms into groups of mutually commuting, and therefore simultaneously measurable, operators. There are a variety of methods through which this grouping can be performed such as mapping to a minimum clique cover problem [70] or using an identity-operator sorting algorithm [25].

In this work, we make use of the fact that only the single-excitation operators need to be measured. For a system represented by N_s spin-orbitals this already limits the number of expectation value measurements to $\mathcal{O}(N_s^2)$. A refinement performed in [21] further reduces the necessary number of measurements to $N_s/2+1$. Here, since the exponentiation of the Hamiltonian scales as $\mathcal{O}(N_s^{16})$ at worst, the $\mathcal{O}(N_s)$ method is not performed and an alternative that scales as $\mathcal{O}(N_s^2)$ but that avoids any need for virtual swapping or circuit recompilation, is employed instead. In comparison to the cost of calculating the moments formulae, the $\mathcal{O}(N_s^2)$ measurement cost is deemed acceptable.

To form TPB groupings the 2-mode operators are first transformed to qubit operators using the Jordan-Wigner transform [60]. For real-valued orbitals and $j<k$;

$$\langle a_j^\dagger a_k\rangle = \frac{1}{4}\big(\big\langle \dots I_{j-1}X_jZ_{j+1}\dots Z_{k-1}X_kI_{k+1}\dots\big\rangle + \big\langle \dots I_{j-1}Y_jZ_{j+1}\dots Z_{k-1}Y_kI_{k+1}\dots\big\rangle\big). \tag{SI.14}$$

For the $j>k$ case, j and k can be exchanged on the right-hand side of the equation. Each 2-mode operator can be characterised by a distance, $d=|j-k|$. By measuring the Pauli strings consisting of X on every qubit and Y on every qubit, all distance, $d=1$, operators can be reconstructed. Likewise measuring the strings $XZXZ\dots$, $YZYZ\dots$, $ZXZX\dots$ and $ZYZY\dots$ allows for the reconstruction of all distance, $d=2$, operators. Continuing this pattern, it can be shown that every $d\in(0,N_s/4]$ operator can be reconstructed by measuring $2d$ strings. For operators with $d\in[N_s/4,N_s/2]$ the operators are less local but there are fewer possible operators and the number of string measurements required is N_s-2d. For the cases considered here, $N_s/4$ is always an integer since the singlet state requires an even number of hydrogen atoms and the spin degeneracy introduces a further factor of 2. In the special case of $d=0$ the expectation value becomes $\langle a_j^\dagger a_j\rangle = \frac{1}{2}(1-\langle Z_j\rangle)$ and can be reconstructed from the four measurements made for the $d=2$ case. The total number of measurements required is then,

$$M = \sum_{d=1}^{N_s/4-1} 2d + \sum_{d=N_s/4}^{N_s/2-1}(N_s-2d) = \frac{N_s^2}{8}, \quad (N_s>4,\ N_s/4\in\mathbb{Z}). \tag{SI.15}$$

Compared to the naive method of measuring each term in the moments formulae individually which scales at worst as $\mathcal{O}(N_s^{16})$, the $\mathcal{O}(N_s^2)$ cost is a significant improvement.

In terms of computation time, the code used to perform the calculations will require further development and optimisation before meaningful comparisons to classical methods can be drawn.

E. RDM purification

In the Hartree-Fock approximation, the one-body reduced density matrix (1-RDM), R, is expected to be idempotent ($R=R^2$). In practice, the presence of errors in the application of gates or in readout, as well as

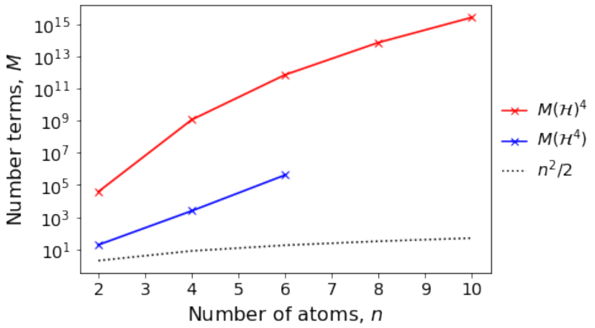

Figure SI.1: Scaling of the number of terms in the fourth moment for the one-dimensional hydrogen chains. The blue line is the observed number of terms in $\mathcal{H}^4$ (before grouping commuting operators) while the red line represents the worst-case scenario of raising the number of terms in $\mathcal{H}$ to the power 4. The dotted black line is the number of measurements required by the method described in Appendix D, $n^2/2$ where n is the number of atoms.

the statistical effects of shot noise lead to the calculated 1-RDM being only nearly idempotent. In this work, the McWeeny iterative purification method [20, 21] is used to correct for these errors. A brief motivation of the procedure is given below;

The error in the RDM (in the sense of its distance from idempotency) can be quantified by $D = R^2 - R$ in which case the objective of the purification is to reduce all elements of the matrix D to 0. By framing this as a minimisation problem with cost function

$$C = \sum_{ij} (D_{ij})^2$$

the RDM can be purified by gradient descent with a step size δ according to;

$$R' = R - \delta \nabla_R(C), \tag{SI.16}$$

where R and R' are the current and updated 1-RDMs respectively and $\nabla_R(C)$ is the gradient of the cost function with respect to the matrix R;

$$\nabla_R(C) = \begin{bmatrix} \frac{\partial C}{\partial R_{11}} & \frac{\partial C}{\partial R_{12}} & \cdots \\ \frac{\partial C}{\partial R_{21}} & \frac{\partial C}{\partial R_{22}} & \cdots \\ \vdots & \vdots & \ddots \end{bmatrix}.$$

In the case of real-valued orbitals, the error matrix D inherits the symmetry of the 1-RDM and so the cost function can be rewritten using the identity

$$\sum_{ij} (D_{ij})^p = \mathrm{Tr}(D^p), \tag{SI.17}$$

for symmetric matrix D and non-negative integer p:

$$C = \mathrm{Tr}(R^4 - 2R^3 + R^2).$$

So the gradient evaluates to

$$\nabla_R(C) = 4R^3 - 6R^2 + 2R.$$

Substituting this into SI.16 and choosing the step size to be $\delta = 1/2$, the expected McWeeny purification formula is recovered

$$R' = 3R^2 - 2R^3. \tag{SI.18}$$

Once the 1-RDM has been measured

$$R = \begin{bmatrix} \langle a_0^\dagger a_0 \rangle & \langle a_0^\dagger a_1 \rangle & \cdots \\ \langle a_1^\dagger a_0 \rangle & \langle a_1^\dagger a_1 \rangle & \cdots \\ \vdots & \vdots & \ddots \end{bmatrix} \tag{SI.19}$$

equation SI.18 can be applied iteratively to obtain a corrected 1-RDM that more accurately represents a single Slater-determinant state. Using the results of Section B, the energy can then be calculated from the elements of this corrected 1-RDM.

F. Sample measurement data

Data for the H_2 molecular Hamiltonian calculated at a bond length of 0.74Åusing the minimal STO-3G basis is presented in table 1. Table 2 contains sample data from the quantum processor *ibmq_sydney* and from which the 1-RDM for molecular hydrogen (with circuit parameter $\theta = 0$) can be constructed. Figure SI.2 presents estimated energies for the H_4 chain for 97 sets of random circuit parameters for both direct and moments-based measurement.

Fermionic operator	$\langle\mathcal{H}\rangle$	$\langle\mathcal{H}^2\rangle$	$\langle\mathcal{H}^3\rangle$	$\langle\mathcal{H}^4\rangle$
$a_0^\dagger a_0$	-1.2533	1.5708	-1.9687	2.4674
$a_1^\dagger a_1$	-0.4751	0.2257	-0.1072	0.0509
$a_2^\dagger a_2$	-1.2533	1.5708	-1.9687	2.4674
$a_3^\dagger a_3$	-0.4751	0.2257	-0.1072	0.0509
$a_1^\dagger a_0^\dagger a_1 a_0$	-0.4825	0.2443	-0.142	0.1089
$a_2^\dagger a_0^\dagger a_2 a_0$	-0.6748	-0.247	2.3385	-6.6903
$a_2^\dagger a_0^\dagger a_3 a_1$	-0.1812	0.3777	-0.7094	1.3164
$a_2^\dagger a_1^\dagger a_2 a_1$	-0.6637	0.6301	-0.7642	1.009
$a_2^\dagger a_1^\dagger a_3 a_0$	-0.1812	0.3859	-0.6222	0.9001
$a_3^\dagger a_0^\dagger a_2 a_1$	-0.1812	0.3859	-0.6222	0.9001
$a_3^\dagger a_0^\dagger a_3 a_0$	-0.6637	0.6301	-0.7642	1.009
$a_3^\dagger a_1^\dagger a_2 a_0$	-0.1812	0.3777	-0.7094	1.3164
$a_3^\dagger a_1^\dagger a_3 a_1$	-0.6977	0.3548	-0.1216	-0.0501
$a_3^\dagger a_2^\dagger a_3 a_2$	-0.4825	0.2443	-0.142	0.1089
$a_2^\dagger a_1^\dagger a_0^\dagger a_2 a_1 a_0$		1.3926	-3.913	8.7428
$a_3^\dagger a_1^\dagger a_0^\dagger a_3 a_1 a_0$		0.6637	-1.1088	1.4847
$a_3^\dagger a_2^\dagger a_0^\dagger a_3 a_2 a_0$		1.3926	-3.913	8.7428
$a_3^\dagger a_2^\dagger a_1^\dagger a_3 a_2 a_1$		0.6637	-1.1088	1.4847
$a_3^\dagger a_2^\dagger a_1^\dagger a_0^\dagger a_3 a_2 a_1 a_0$		2.4195	-5.4784	10.9158

Table SI.1: Fermionic operator weights for molecular hydrogen calculated in the STO-3G basis at equilibrium bond length (0.74Å). Molecular orbitals are ordered from 0 to 3 as spin-up bonding, spin-up anti-bonding, spin-down bonding, spin-down anti-bonding

$\langle a_i^\dagger a_j\rangle$	$j=0,2$	$j=1,3$
$i=0,2$	0.96	-0.0114
$i=1,3$	-0.0114	0.0236

$\langle a_i^\dagger a_j\rangle$	$j=0,2$	$j=1,3$
$i=0,2$	0.9619	-0.0082
$i=1,3$	-0.0082	0.0229

$\langle a_i^\dagger a_j\rangle$	$j=0,2$	$j=1,3$
$i=0,2$	0.9647	-0.0036
$i=1,3$	-0.0036	0.023

$\langle a_i^\dagger a_j\rangle$	$j=0,2$	$j=1,3$
$i=0,2$	0.9675	-0.0095
$i=1,3$	-0.0095	0.0262

Table SI.2: Four sets of 1-RDM elements for the hydrogen molecule ($\theta = 0$) calculated on *ibmq_sydney* with ≈ 2000 shots each. The ordering of orbitals matches that used in table 1.

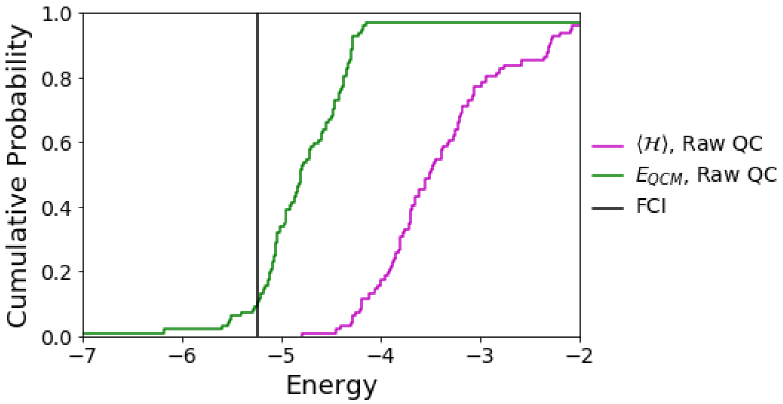

Figure SI.2: Cumulative frequency plot for the H_4 molecular chain. 100 sets of uniform random parameters were chosen and the energy was evaluated both directly and by the QCM method on the quantum processor *ibmq_montreal*. The vertical axis is the cumulative frequency, i.e. the fraction of points with a measured energy below a given value. In the ideal case (that the FCI energy is recovered regardless of the parameter values) the resulting plot would be the step function at the FCI energy (black line). Of the 100 random points, only 97 are represented in the plot. The remaining 3 points were rejected due to large variations in the QCM energy when repeated, e.g. standard deviations of the same scale or larger than the energy measurements. These anomalous results are likely due to the combination of a poorly conditioned point and errors in the device leading to non-physical density matrices. The large deviations are seen to vanish when McWeeny purification is applied.

[1] R. P. Feynman, "Simulating physics with computers," *International Journal of Theoretical Physics*, vol. 21, no. 6, pp. 467–488, 1982.

[2] M. Reiher, N. Wiebe, K. M. Svore, D. Wecker, and M. Troyer, "Elucidating reaction mechanisms on quantum computers," *Proceedings of the National Academy of Sciences*, vol. 114, no. 29, pp. 7555–7560, 2017.

[3] J. M. Montgomery and D. A. Mazziotti, "Strong electron correlation in nitrogenase cofactor, FeMoco," *The Journal of Physical Chemistry A*, vol. 122, pp. 4988–4996, Jun 2018.

[4] V. E. Elfving, B. W. Broer, M. Webber, J. Gavartin, M. D. Halls, K. P. Lorton, and A. Bochevarov, "How will quantum computers provide an industrially relevant computational advantage in quantum chemistry?." arXiv:quant-ph/2009.12472, 2020.

[5] V. von Burg, G. H. Low, T. Häner, D. S. Steiger, M. Reiher, M. Roetteler, and M. Troyer, "Quantum computing enhanced computational catalysis," *Phys. Rev. Research*, vol. 3, p. 033055, Jul 2021.

[6] D. Wecker, B. Bauer, B. K. Clark, M. B. Hastings, and M. Troyer, "Gate-count estimates for performing quantum chemistry on small quantum computers," *Phys. Rev. A*, vol. 90, p. 022305, Aug 2014.

[7] I. D. Kivlichan *et al.*, "Improved fault-tolerant quantum simulation of condensed-phase correlated electrons via Trotterization," *Quantum*, vol. 4, p. 296, 2020.

[8] A. Peruzzo, J. McClean, P. Shadbolt, M.-H. Yung, X.-Q. Zhou, P. J. Love, A. Aspuru-Guzik, and J. L. O'Brien, "A variational eigenvalue solver on a photonic quantum processor," *Nature Communications*, vol. 5, no. 1, p. 4213, 2014.

[9] A. Kandala, A. Mezzacapo, K. Temme, M. Takita, M. Brink, J. M. Chow, and J. M. Gambetta, "Hardware-efficient variational quantum eigensolver for small molecules and quantum magnets," *Nature*, vol. 549, no. 7671, pp. 242–246, 2017.

[10] M. Cerezo, A. Arrasmith, R. Babbush, S. C. Benjamin, S. Endo, K. Fujii, J. R. McClean, K. Mitarai, X. Yuan, L. Cincio, and P. J. Coles, "Variational quantum algorithms," *Nature Reviews Physics*, vol. 3, no. 9, pp. 625–644, 2021.

[11] J. Preskill, "Quantum computing and the entanglement frontier." arXiv:quant-ph/1203.5813, 2012.

[12] Y. Shen, X. Zhang, S. Zhang, J.-N. Zhang, M.-H. Yung, and K. Kim, "Quantum implementation of the unitary coupled cluster for simulating molecular electronic structure," *Phys. Rev. A*, vol. 95, p. 020501, Feb 2017.

[13] P. J. J. O'Malley, R. Babbush, *et al.*, "Scalable quantum simulation of molecular energies," *Phys. Rev. X*, vol. 6, p. 031007, Jul 2016.

[14] H. R. Grimsley, S. E. Economou, E. Barnes, and N. J. Mayhall, "An adaptive variational algorithm for exact molecular simulations on a quantum computer," *Nature Communications*, vol. 10, no. 1, p. 3007, 2019.

[15] H. L. Tang *et al.*, "Qubit-ADAPT-VQE: An adaptive algorithm for constructing hardware-efficient ansätze on a quantum processor," *PRX Quantum*, vol. 2, p. 020310, Apr 2021.

[16] R. Santagati *et al.*, "Witnessing eigenstates for quantum simulation of Hamiltonian spectra," *Science Advances*, vol. 4, no. 1, 2018.

[17] J. Preskill, "Quantum computing in the NISQ era and beyond," *Quantum*, vol. 2, p. 79, 2018.

[18] L. F. Richardson and J. A. Gaunt, "The deferred approach to the limit," *Philosophical Transactions of the Royal Society of London*, vol. 226, Jan 1927.

[19] A. Kandala, K. Temme, A. D. Córcoles, A. Mezzacapo, J. M. Chow, and J. M. Gambetta, "Error mitigation extends the computational reach of a noisy quantum processor," *Nature*, vol. 567, no. 7749, pp. 491–495, 2019.

[20] R. McWeeny, "Some recent advances in density matrix theory," *Rev. Mod. Phys.*, vol. 32, pp. 335–369, Apr 1960.

[21] F. Arute *et al.*, "Hartree-Fock on a superconducting qubit quantum computer," *Science*, vol. 369, no. 6507, pp. 1084–1089, 2020.

[22] D. Thouless, "Stability conditions and nuclear rotations in the Hartree-Fock theory," *Nuclear Physics*, vol. 21, pp. 225 – 232, 1960.

[23] A. G. Taube and R. J. Bartlett, "New perspectives on unitary coupled-cluster theory," *International Journal of Quantum Chemistry*, vol. 106, no. 15, pp. 3393–3401, 2006.

[24] J. Lee, W. J. Huggins, M. Head-Gordon, and K. B. Whaley, "Generalized unitary coupled cluster wave functions for quantum computation," *Journal of Chemical Theory and Computation*, vol. 15, pp. 311–324, Jan 2019.

[25] H. J. Vallury, M. A. Jones, C. D. Hill, and L. C. L. Hollenberg, "Quantum computed moments correction to variational estimates," *Quantum*, vol. 4, p. 373, Dec 2020.

[26] L. C. L. Hollenberg, "Plaquette expansion in lattice Hamiltonian models," *Phys. Rev. D*, vol. 47, pp. 1640–1644, Feb 1993.

[27] K. Seki and S. Yunoki, "Quantum power method by a superposition of time-evolved states," *PRX Quantum*, vol. 2, p. 010333, Feb 2021.

[28] P. Suchsland, F. Tacchino, M. H. Fischer, T. Neupert, P. K. Barkoutsos, and I. Tavernelli, "Algorithmic error mitigation scheme for current quantum processors," *Quantum*, vol. 5, p. 492, 2021.

[29] K. Kowalski and B. Peng, "Quantum simulations employing connected moments expansions," *The Journal of Chemical Physics*, vol. 153, no. 20, p. 201102, 2020.

[30] B. Peng and K. Kowalski, "Variational quantum solver employing the PDS energy functional," *Quantum*, vol. 5, p. 473, 2021.

[31] D. Claudino, B. Peng, N. Bauman, K. Kowalski, and T. S. Humble, "Improving the accuracy and efficiency of quantum connected moments expansions," *Quantum Science and Technology*, 2021.

[32] J. Cioslowski, "Connected moments expansion: A new tool for quantum many-body theory," *Phys. Rev. Lett.*, vol. 58, pp. 83–85, Jan 1987.

[33] L. C. L. Hollenberg and N. S. Witte, "Analytic solution for the ground-state energy of the extensive many-body problem," *Phys. Rev. B*, vol. 54, pp. 16309–16312, 1996.

[34] J. R. McClean, M. E. Kimchi-Schwartz, J. Carter, and W. A. de Jong, "Hybrid quantum-classical hierarchy for mitigation of decoherence and determination of excited states," *Phys. Rev. A*, vol. 95, p. 042308, Apr 2017.

[35] T. Takeshita, N. C. Rubin, Z. Jiang, E. Lee, R. Babbush, and J. R. McClean, "Increasing the representation accuracy of quantum simulations of chemistry without extra quantum resources," *Phys. Rev. X*, vol. 10, p. 011004, Jan 2020.

[36] J. I. Colless *et al.*, "Computation of molecular spectra on a quantum processor with an error-resilient algorithm," *Phys. Rev. X*, vol. 8, p. 011021, Feb 2018.

[37] M. Motta *et al.*, "Determining eigenstates and thermal states on a quantum computer using quantum imaginary time evolution," *Nature Physics*, vol. 16, no. 2, pp. 205–210, 2020.

[38] K. Yeter-Aydeniz, R. C. Pooser, and G. Siopsis, "Practical quantum computation of chemical and nuclear energy levels using quantum imaginary time evolution and Lanczos algorithms," *npj Quantum Information*, vol. 6, no. 1, p. 63, 2020.

[39] W. J. Huggins, J. Lee, U. Baek, B. O'Gorman, and K. B. Whaley, "A non-orthogonal variational quantum eigensolver," *New Journal of Physics*, vol. 22, p. 073009, Jul 2020.

[40] N. H. Stair, R. Huang, and F. A. Evangelista, "A multireference quantum Krylov algorithm for strongly correlated electrons," *Journal of Chemical Theory and Computation*, vol. 16, no. 4, pp. 2236–2245, 2020. PMID: 32091895.

[41] R. M. Parrish and P. L. McMahon, "Quantum filter diagonalization: Quantum eigendecomposition without full quantum phase estimation." arXiv:quant-ph/1909.08925, 2019.

[42] J. Cohn, M. Motta, and R. M. Parrish, "Quantum filter diagonalization with double-factorized Hamiltonians." arXiv:quant-ph/2104.08957, 2021.

[43] L. C. L. Hollenberg, M. P. Wilson, and N. S. Witte, "General nonperturbative mass gap to first order in $1/V$," *Phys. Lett. B*, vol. 361, pp. 81–86, 1995.

[44] I. Kassal and A. Aspuru-Guzik, "Quantum algorithm for molecular properties and geometry optimization," *The Journal of Chemical Physics*, vol. 131, no. 22, p. 224102, 2009.

[45] F. Jamet, A. Agarwal, C. Lupo, D. E. Browne, C. Weber, and I. Rungger, "Krylov variational quantum algorithm for first principles materials simulations." arXiv:quant-ph/2105.13298, 2021.

[46] E. A. R. Guzman and D. Lacroix, "Predicting ground state, excited states and long-time evolution of many-body systems from short-time evolution on a quantum computer." arXiv:quant-ph/2104.08181, 2021.

[47] D. Wecker, M. B. Hastings, N. Wiebe, B. K. Clark, C. Nayak, and M. Troyer, "Solving strongly correlated electron models on a quantum computer," *Phys. Rev. A*, vol. 92, p. 062318, Dec 2015.

[48] I. D. Kivlichan, J. McClean, N. Wiebe, C. Gidney, A. Aspuru-Guzik, G. K.-L. Chan, and R. Babbush, "Quantum simulation of electronic structure with linear depth and connectivity," *Phys. Rev. Lett.*, vol. 120, p. 110501, Mar 2018.

[49] J. M. Arrazola, O. D. Matteo, N. Quesada, S. Jahangiri, A. Delgado, and N. Killoran, "Universal quantum circuits for quantum chemistry." arXiv:quant-ph/2106.13839, 2021.

[50] "Quantum user interface tool." quispace.org, (2018-2020).

[51] A. Y. Kitaev, "Quantum measurements and the abelian stabilizer problem." arXiv:quant-ph/9511026, 1995.

[52] A. Duan, "Matrix product states in quantum information processing," Master's thesis, University of Melbourne, Australia, 2015.

[53] M. A. Jones, "Moments-based corrections to variational quantum computation," Master's thesis, University of Melbourne, Australia, 2019.

[54] D. Horn and M. Weinstein, "The t expansion: A nonperturbative analytic tool for Hamiltonian systems," *Phys. Rev. D*, vol. 30, pp. 1256–1270, Sep 1984.

[55] L. C. L. Hollenberg and N. S. Witte, "General nonperturbative estimate of the energy density of lattice Hamiltonians," *Phys. Rev. D*, vol. 50, pp. 3382–3386, Sep 1994.

[56] Q. Sun *et al.*, “Pyscf: the python-based simulations of chemistry framework,” *Wiley Interdisciplinary Reviews: Computational Molecular Science*, vol. 8, no. 1, p. e1340, 2017.

[57] Y. Kawashima, E. Lloyd, M. P. Coons, Y. Nam, S. Matsuura, A. J. Garza, S. Johri, L. Huntington, V. Senicourt, A. O. Maksymov, J. H. V. Nguyen, J. Kim, N. Alidoust, A. Zaribafiyan, and T. Yamazaki, “Optimizing electronic structure simulations on a trapped-ion quantum computer using problem decomposition,” *Nature Communications Physics*, vol. 4, no. 1, p. 245, 2021.

[58] A. J. McCaskey, Z. P. Parks, J. Jakowski, S. V. Moore, T. D. Morris, T. S. Humble, and R. C. Pooser, “Quantum chemistry as a benchmark for near-term quantum computers,” *npj Quantum Information*, vol. 5, no. 1, p. 99, 2019.

[59] Y. Nam *et al.*, “Ground-state energy estimation of the water molecule on a trapped-ion quantum computer,” *npj Quantum Information*, vol. 6, no. 1, p. 33, 2020.

[60] P. Jordan and E. Wigner, “Über das paulische äquivalenzverbot,” *Zeitschrift für Physik*, vol. 47, no. 9, pp. 631–651, 1928.

[61] L. Lafayette, G. Sauter, L. Vu, and B. Meade, “Spartan performance and flexibility: An HPC-cloud chimera.” OpenStack Summit, Barcelona, doi.org/10.4225/49/58ead90dceaaa, Oct 2016.

[62] C. Lanczos, “An iteration method for the solution of the eigenvalue problem of linear differential and integral operators,” *J. Res. Natl. Bur. Stand. B*, vol. 45, pp. 255–282, 1950.

[63] L. C. L. Hollenberg, D. C. Bardos, and N. S. Witte, “Lanczos cluster expansion for non-extensive systems,” *Zeitschrift für Physik D Atoms, Molecules and Clusters*, vol. 38, no. 3, pp. 249–252, 1996.

[64] S. Bravyi, J. M. Gambetta, A. Mezzacapo, and K. Temme, “Tapering off qubits to simulate fermionic hamiltonians.” arXiv:quant-ph/1701.08213, 2017.

[65] P. A. M. Dirac, “Note on the interpretation of the density matrix in the many-electron problem,” *Proc. Cambridge Phil. Soc.*, vol. 27, no. 2, pp. 240–243, 1931.

[66] H. Abraham *et al.*, “Qiskit: An open-source framework for quantum computing,” 2019.

[67] R. Babbush, N. Wiebe, J. McClean, J. McClain, H. Neven, and G. K.-L. Chan, “Low-depth quantum simulation of materials,” *Phys. Rev. X*, vol. 8, p. 011044, Mar 2018.

[68] R. Babbush, D. W. Berry, J. R. McClean, and H. Neven, “Quantum simulation of chemistry with sublinear scaling in basis size,” *npj Quantum Information*, vol. 5, no. 1, p. 92, 2019.

[69] J. S. Kottmann *et al.*, “Reducing qubit requirements while maintaining numerical precision for the variational quantum eigensolver: A basis-set-free approach,” *The Journal of Physical Chemistry Letters*, vol. 12, pp. 663–673, Jan 2021.

[70] V. Verteletskyi, T.-C. Yen, and A. F. Izmaylov, “Measurement optimization in the variational quantum eigensolver using a minimum clique cover,” *The Journal of Chemical Physics*, vol. 152, no. 12, p. 124114, 2020.
